# STRUCTURAL STRESSORS, PERCEIVED NEIGHBORHOOD CONTEXT, AND COGNITIVE FUNCTION IN BLACK MIDLIFE AND OLDER ADULTS

**DOI:** 10.1093/geroni/igae098.1306

**Published:** 2024-12-31

**Authors:** Amy Thierry, Kazumi Tsuchiya, Harry Taylor

**Affiliations:** Xavier University of Louisiana, New Orleans, Louisiana, United States; University of Toronto, Toronto, Ontario, Canada; University of Toronto, Toronto, Ontario, Canada

## Abstract

Racial disparities in Alzheimer’s disease and related dementias have been established in the US, with higher rates in Black compared to White older adults. Because Black populations are disproportionately exposed to greater neighborhood disadvantage, chronic stress via the neighborhood context may contribute to worse cognitive health among Black older adults. However, the role of neighborhood-based stressors on cognitive function within the Black population is less understood. This study aims to examine how lifetime exposure to structural stressors (e.g., discrimination in housing, unemployment, and policing) and current perceived neighborhood stressors (e.g., high disorder and social discohesion) independently and interactively are associated with cognitive function in Black adults >50 years of age (n=1,663) using 2010-2016 Health and Retirement Study data. Linear regression models tested associations between structural stressors, perceived neighborhood stressors, and interaction terms of total number of structural stressors, neighborhood disorder (including safety and cleanliness), and social discohesion with cognitive function measured using the Langa-Weir 27-point scale. Black adults reported exposure to structural stressors as 15.69% in policing, 14.25% in hiring, and 7.88% in housing. In unadjusted models, greater total structural stressors were associated with greater cognitive function, while worse ratings of social cohesion, safety and cleanliness were associated with lower cognitive function. A statistically significant interaction term (b= -.221, SE=0.10, p<0.05) indicated that high social discohesion and greater total structural stressors was associated with lower cognitive function. Thus, life course exposure to multiple domains of neighborhood-based stressors may be important for Black adults’ cognitive function in later life.

